# How free-ranging ungulates with differing water dependencies cope with seasonal variation in temperature and aridity

**DOI:** 10.1093/conphys/coz064

**Published:** 2019-11-07

**Authors:** Melinda Boyers, Francesca Parrini, Norman Owen-Smith, Barend F N Erasmus, Robyn S Hetem

**Affiliations:** 1 Centre for African Ecology, School of Animal, Plant and Environmental Sciences, University of the Witwatersrand, Jan Smuts Avenue, Braamfontein 2000, Johannesburg, South Africa; 2 Global Change Institute, University of the Witwatersrand, Jan Smuts Avenue, Braamfontein 2000, Johannesburg, South Africa; 3 Brain Function Research Group, School of Physiology, University of the Witwatersrand, Jan Smuts Avenue, Braamfontein 2000, Johannesburg, South Africa; 4 School of Animal, Plant and Environmental Sciences, University of the Witwatersrand, Jan Smuts Avenue, Braamfontein 2000, Johannesburg, South Africa

**Keywords:** Aridity, behaviour flexibility, biologging, Climate Change, thermoregulation

## Abstract

Large mammals respond to seasonal changes in temperature and precipitation by behavioural and physiological flexibility. These responses are likely to differ between species with differing water dependencies. We used biologgers to contrast the seasonal differences in activity patterns, microclimate selection, distance to potential water source and body temperature of the water-independent gemsbok (*Oryx gazella gazella*) and water-dependent blue wildebeest (*Connochaetes taurinus*), free-living in the arid Kalahari region of Botswana. Gemsbok were more active nocturnally during the hot seasons than in the cold-dry season, while wildebeest showed no seasonal difference in their nocturnal activity level. Both species similarly selected shaded microclimates during the heat of the day, particularly during the hot seasons. Wildebeest were further than 10 km from surface water 30% or more of the time, while gemsbok were frequently recorded >20 km from potential water sources. In general, both species showed similar body temperature variation with high maximum 24-h body temperature when conditions were hot and low minimum 24-h body temperatures when conditions were dry, resulting in the largest amplitude of 24-h body temperature rhythm during the hot-dry period. Wildebeest thus coped almost as well as gemsbok with the fairly typical seasonal conditions that occurred during our study period. They do need to access surface water and may travel long distances to do so when local water sources become depleted during drought conditions. Thus, perennial water sources should be provided judiciously and only where essential.

## Teaser

**Lay summary**: We compared the behavioural and physiological flexibility of two sympatric species with different water dependencies to seasonal variation in temperature and aridity. Both species coped relatively well with current fluctuations, but as water sources become less reliable under future climate change scenarios, the water-dependent species may risk extirpation.

## Introduction

Climate change is already affecting numerous organisms (Parmesan and Yohe, 2003; Pio *et al.*, 2014). Rising global temperatures will place increasing stress on free-ranging wild animals, either directly through heat tolerance or indirectly through changes in resources (Fuller *et al.*, 2016). Semi-arid savannas of southern Africa already represent a highly stressful environment with limited water resources and extreme temperatures (Bergström and Skarpe, 1999; Makhabu *et al.*, 2002), with rainfall generally restricted to summer months. When rains are delayed at the start of the wet season, species are concurrently exposed to high ambient temperatures, poor forage quality and limited access to water during a time when their body reserves are low (Fynn, 2012; Owen-Smith, 2008). Behavioural flexibility is likely to be an animal’s primary response to changes in climatic conditions (Hetem *et al.*, 2014), with responses likely to be different between water-dependent and water-independent species.

Animals may alleviate radiant heat loads during the day by seeking shade, at the cost of diurnal foraging activity (Fuller *et al.*, 2016). They may compensate to some extent by foraging more at night (Hetem *et al.*, 2012b), thereby elevating their exposure to predation by nocturnal carnivores (Owen-Smith, 2015). Water-dependent species become restricted to commuting distance from long-lasting water sources during the dry season, restricting their access to food (Western, 1975). Heterothermy, variability in body temperature beyond the limits of homeothermy (IUPS Thermal Commision, 2001), may provide an index of physiological well-being (Hetem *et al.*, 2016; Maloney *et al.*, 2017). When water is accessible, large ungulates can use evaporative cooling to dissipate body heat when thermal heat loads are high (Taylor, 1970). However, when access to water is limited, ungulates must ‘trade off’ osmoregulation (control of body water) with thermoregulation (control of body temperature; Hetem *et al*., 2016) and an increase in maximum 24-h body temperatures may result (Hetem *et al.*, 2010; Tattersall *et al.*, 2012). Yet, it is not only water stress that affects thermoregulation; food deficiencies may also restrict the animal ability to maintain body temperature (Hetem *et al.*, 2016). If forage quality and quantity are limited or individuals are unable to compensate for reduced diurnal foraging, a negative energy balance may result in a drop in minimum 24-h body temperature (Hetem *et al.*, 2016; Maloney *et al.*, 2011; Tattersall *et al.*, 2012). Therefore, understanding the physiological and behavioural flexibility of individuals currently inhabiting hot and dry environments is crucial to improving predictions about how species might respond to hotter and drier environments predicted for arid and semi-arid regions in Africa under climate change (Engelbrecht *et al.*, 2015).

The Kalahari region of southern Africa represents a particularly hot and dry environment with very little surface water, while air temperatures can exceed 40°C in summer and frequently drop below freezing in winter (Knight, 1991). Nevertheless, this region supports a variety of large mammals, including the arid-adapted gemsbok (*Oryx gazella gazella*) and the more water-dependent blue wildebeest (*Connochaetes taurinus*). Gemsbok seem able to survive without access to drinking water (Taylor, 1969). They have the ability to reabsorb more water from long Loops of Henle in their kidneys and absorb more water from the colon compared to wildebeest, thereby producing more concentrated urine and drier dung than wildebeest (Knight, 2013; Van Hoven, 1983). In addition, based on a single laboratory study, gemsbok have been reported to employ heterothermy to survive high heat loads (Taylor, 1969), whereas free-living gemsbok with free access to water maintained homeothermy (Maloney *et al.*, 2002). Free-living wildebeest, on the other hand, reportedly drink every other day when grass becomes dry (Berry, 1980) and may show progressive lowering of minimum 24-h body temperature throughout the dry season (Shrestha *et al.*, 2012).

The aim of this paper is therefore to give an overview of the seasonal variations in activity, microclimate selection, spatial location and body temperature, comparing free-living water-independent gemsbok with similarly sized water-dependent blue wildebeest over the same seasonal cycle. We expected that, compared with gemsbok, wildebeest would (i) show a greater relative increase in nocturnal activity during the hot seasons and (ii) seek cooler microclimates to a greater extent during the heat of the day, while (iii) remaining closer to surface water during the dry seasons and, when unable to cope with the thermal and water stress, (iv) display more variable body temperatures during the hot-dry season.

## Materials and methods

### Animals and study area

The study took place between August 2013 and May 2015 within south-western Botswana, encompassing the Kgalagadi Transfrontier Park (Botswana side) and adjoining wildlife management areas known as the Bakgalagadi Schwelle region (S 24.35°, E 20.62°). This area consists primarily of open woodland, overlying a sandy substrate with no permanent natural water sources. Thus, herbivores depend on ephemeral pools that form shortly after rain and a few artificial (i.e. manmade) waterholes with differing degrees of potability (i.e. not all are suitable for drinking). Two artificially maintained water points occurred within our study area at Kaa gate in the south-west and Zutshwa Village water reservoir in the east. Nossob River lies in the south with perennially maintained water. Numerous pan depressions retained water for variable periods after rain.

Common plant species include *Vachellia erioloba*, *Vachellia luederitzii*, *Boscia albitrunca*, *Terminalia sericea*, *Grewia flava* and *Senegalia mellifera* among trees and *Schmidtia kalahariensis* and *Stipagrostis uniplumis* in the grass layer on sandy substrates (Keeping, 2014; van Rooyen *et al.*, 2008). *Vachellia erioloba*, in particular, retained leaves over the seasonal cycle within our study area, which provides vital shade during the dry season when many other trees are leafless (Barnes et al., 1997). Large carnivores included lion (*Panthera leo*), leopard (*Panthera pardus*) and spotted hyena (*Crocuta crocuta*), while common herbivores included red hartebeest (*Alcelaphus buselaphus caama*), eland (*Tragelaphus oryx*) and springbok (*Antidorcas marsupialis*), in addition to gemsbok and blue wildebeest. Population estimates for the Kgalagadi District were around 90 000 gemsbok and 9500 blue wildebeest (Statistics Botswana, 2014).

Eight individual female gemsbok and eight individual female blue wildebeest, each in distinct herds, were captured in August 2013 to attach and implant biologgers. The Government of Botswana via the Ministry of Environment, Wildlife and Tourism and Department of Wildlife and National Parks granted approvals and permits [numbers EWT 8/36/4 XX (32), EWT 8/36/4 XXVII (15), EWT 8/36/4 XXIV (102)] to conduct the study. The Animal Ethics Screening Committee of the University of the Witwatersrand (protocol no. 2012/24/04) approved all experimental procedures.

### Surgical procedure

All 16 animals were darted from a helicopter by an experienced veterinarian. Each dart contained a combination of thiafentanil (gemsbok: 7–8 mg, wildebeest: 4–6 mg, Thianil, Kyron Laboratories, Johannesburg, South Africa), medetomidine hydrochloride (gemsbok: 3–6 mg, wildebeest: 2–4 mg, medetomidine, Kyron Laboratories, Johannesburg, South Africa) and ketamine (gemsbok: 75–150 mg, wildebeest: 50–150 mg, Pfizer Animal Health, Sandton, South Africa). Once the animal was immobile, we placed it into sternal recumbency, supported by sandbags, with head elevated. We intubated the animals, and anaesthesia was maintained with 2–5% isoflurane (AErrane, Astra Zeneca, Johannesburg, South Africa), administered in 100% oxygen. Throughout the surgery, which lasted approximately 30–45 min, respiratory rate, heart rate, arterial oxygen saturation and rectal temperature were monitored.

We shaved and sterilised the incision sites with chlorhexidine gluconate (Hibitane, Zeneca, Johannesburg, South Africa) and injected a local anaesthetic (3 mL 2% subcutaneously (S.C.); lignocaine hydrochloride, Bayer Animal Health (Pty) Ltd, Isando, South Africa). We placed the miniature temperature-sensitive data loggers in the retroperitoneal space and tethered the motion-sensitive data loggers to the abdominal muscle wall. Once the loggers were in place, the muscle and skin layers were sutured closed. Wounds were sprayed with a topical antiseptic spray (Necrospray, Bayer (Pty) Ltd, Isando, South Africa) and coated with tick grease (SWAVET RSA (Pty) Ltd, Northriding, South Africa). Each animal received a long-acting antibiotic (~0.04 mL kg^−1^, intramuscularly (I.M.), penicillin, Duplocillin LA, Schering-Plough Animal Health Ltd, New Zealand) and a non-steroidal anti-inflammatory analgesic (~0.5 mg kg^−1^ I.M., Metacam, meloxicam-injectable solution, Boehringer Ingelheim Vetmedica, Inc., St. Joseph, USA).

A neck collar (African Wildlife Tracking, Pretoria, South Africa) containing a miniglobe thermometer, a satellite-linked global positioning system (GPS) data logger, and a very high frequency (VHF) radiotelemetry beacon was fitted to each animal. After surgery, inhalation anaesthesia was terminated, and the immobilising drug was completely reversed by a combination of naltrexone (gemsbok: 75–120 mg, wildebeest: 60–100 mg, I.M. Naltrexone, Kyron Laboratories, Johannesburg, South Africa) and atipamezole (gemsbok: 10–20 mg; wildebeest: 10–15 mg, I.M. Antisedan, atipamezole hydrochloride, Orion Corporation, Orion Pharma, Finland). As soon as the animal was awake and mobile, it was left undisturbed to roam freely, until recapture to retrieve the collars and loggers at the end of the study period.

### Temperature and activity measurements

Each GPS collar included a 12-h on/off VHF radiotelemetry beacon. In addition, each collar supported a miniature black globe thermometer (‘miniglobe’). A miniglobe is a small (30-mm-diameter) hollow copper sphere painted matt black with a temperature sensor inside it. This miniglobe, attached to the top of the collar, recorded the thermal microclimate that the animal occupied (Hetem *et al.*, 2007). Miniglobe temperature provides a better index of heat stress than air temperature as it integrates the effects of air temperature, radiation and wind speed. Miniglobe temperatures were recorded hourly and relayed back via satellite. These miniglobe’s temperature sensors had a resolution and calibrated accuracy of 0.5°C and a measurement range from −10 to 85°C. The total mass of each collar was ~1.2 kg, which was ~0.6% of the animal’s body mass.

The miniature temperature-sensitive data loggers (DST Centi-T, Star-Oddi, Iceland) had outside dimensions of ~15 × 46 mm (diameter × length) and a mass of 20 g. They were covered with biologically and chemically inert wax (Sasol, Johannesburg, South Africa) and sterilised using an instant sterilant (F10 Sterilant with rust inhibitor, Health and Hygiene (Pty) Ltd, Roodepoort, South Africa) before implantation. Recorded data was stored onboard at a resolution of 0.03°C and measurement range from 5 to 45°C and calibrated accuracy better than 0.1°C. The scan interval of the temperature-sensitive loggers was 10 min. All temperature sensors and loggers were calibrated against a high-accuracy thermometer (Quat 100, Heraeus, Hanau, Germany) in an insulated water bath.

The motion-sensitive loggers (ADXL345, Sigma Delta Technologies, Australia) recorded movement at 5-min intervals, had dimensions of 35 × 35 × 10 mm and weighed 20 g when covered in wax. The motion-sensitive logger had a triaxial accelerometer with equal sensitivity across three planes (resolution one-fourth 4 mg/least significant bit), and motion changes were recorded as activity counts within the first 10 s of each 5-min interval; similar intervals at the start of every minute were validated against behavioural observations of vervet monkeys (*Chlorocebus pygerythrus*; McFarland *et al*., 2013). To account for differences in the sensitivity of individual motion-sensitive loggers, we calculated activity as a percentage of the maximum activity reading that each logger recorded, over the entire study period. The total mass of equipment implanted and attached to the animals was less than 1% of their body mass and is unlikely to have adversely affected their behaviour.

### Weather data measurements

We recorded black globe temperature (HOBO Weather Logger [H21-001], Onset Computer Corporation, USA) hourly at Kaa gate, Kgalagadi Transfrontier Park (S 24.3451°, E 20.6049°). A free-standing miniglobe thermometer, identical to the collar miniglobe thermometer, was placed in direct sun, 1 m aboveground, and recorded temperature (°C) every hour (reference miniglobe). Daily rainfall (mm) data were obtained from Climate Hazards group InfraRed Precipitation with Station data (CHIRPS) for the same location as the weather station (S 24.3451°, E 20.6049°). In order to define seasonal periods, daily vegetation greenness data were obtained from the MODIS Terra Daily Normalised Difference Vegetative Index (NDVI) dataset averaged across the entire study area. Green plants have high reflectivity in the near-infrared (NIR) wavelengths and absorb red wavelengths for photosynthesis, which results in an NDVI ratio that ranges from −1 to +1, where negative values correspond to an absence of vegetation (i.e. soil; Myneni *et al*., 1995). Potential surface water was identified from Landsat imagery as per method established in Boyers (2018).

### Data analysis

The data collection covered 21 months, from August 2013 until May 2015. Two wildebeest collars stopped sending data 2 weeks after placement and could not be relocated. Three of the gemsbok collars stopped sending data in October 2013. The VHF beacon allowed us to locate the carcass remains of the three gemsbok, but no data loggers were found. Twenty-one months after the initial capture, in May 2015, the 11 remaining animals were located via the VHF beacons and darted from a helicopter. As per the original surgery, the animals were anaesthetized, and data loggers were surgically removed. Thereafter, the animals were released. However, many of the GPS units and activity loggers stopped working after December 2014; therefore, we focussed our analyses on the annual cycle between December 2013 and November 2014 using data provided by 11 (five gemsbok and six wildebeest) body temperature loggers; six (three gemsbok and three wildebeest) activity loggers; seven (five gemsbok and two wildebeest) miniglobe temperature sensors; and seven (five gemsbok and two wildebeest) GPS units.

For seasonal analysis, we subdivided data into four seasonal blocks consisting of 3 months each: ‘hot-wet’ (December to February), ‘transition-dry’ (March to May), ‘cold-dry’ (June to August) and ‘hot-dry’ (September to November). These seasonal blocks allowed us to compare two hot seasons, which differed in rainfall and aridity (assessed by NDVI) but not temperature.

To assess the 24-h rhythm of activity, we summed activity per hour per individual and averaged these across each season per species. We compared the proportion of total 24-h activity that occurred during the nocturnal hours (sunset to sunrise) between species and across seasons with a two-way repeated measures ANOVA with individual animals as replicates and Tukey’s multiple comparison post hoc test. Proportion data were arcsine-transformed to comply with test assumptions.

To quantify microclimate selection, we calculated the difference between miniglobe temperature on the collar and the reference miniglobe located in the sun per individual per hour. To assess the 24-h rhythm of microclimate selection, we calculated the mean and SD of the temperature differences per hour across each season per species. We also calculated the minimum 24-h difference between the collar and reference miniglobe temperature (i.e. the coolest microclimate sought) and defined it as ‘intensity of shade’. To assess the average temperature at which each individual sought shade, we correlated collar miniglobe temperatures against reference miniglobe temperatures per individual using non-linear polynomial regression and derived the temperature at which the regression line intersected with the line of identity (where the *x*-axis equalled the *y*-axis), which was defined as ‘threshold temperature’ (see Fig. 3). Both the intensity of shade and the threshold temperatures were compared between species and across seasons with two-way repeated measures ANOVAs with individual animals as replicates and Tukey’s multiple comparisons post hoc test.

To assess the spatial distribution relative to potential water sources, defined as ‘distance to water’, we calculated the minimum 24-h distance to a potential water source, either pan with water or artificial water hole, from the hourly GPS locations from the collared individuals, as described in Boyers (2018). We averaged the minimum 24-h distance to water for each individual, per season and compared species across seasons with a two-way repeated measures ANOVA with individual animals as replicates and Tukey’s multiple comparisons post hoc tests. We also classified the distance to water (as calculated above) into five categories, namely 0–2, 2–5, 5–10, 10–20 and >20 km and calculated the proportion of time spent in each category per individual averaged across seasons.

To assess the 24-h rhythm of body temperature, we averaged 10-min recordings of body temperature per individual and averaged across each season per species. For successive 24-h periods, we calculated the mean, minimum, maximum and amplitude (maximum minus minimum) of body temperature rhythm for each individual animal. We averaged body temperature parameters for each individual, for each season, and compared species across seasons with a two-way repeated measures ANOVA with individual animals as replicates and Tukey’s multiple comparisons post hoc tests.

Statistical analyses were performed using GraphPad Prism (version 6.00 for Windows, GraphPad Software, San Diego, CA, USA). Values are expressed as mean and standard deviation, and *P* < 0.05 was considered significant. All reported ANOVA results met the assumptions of homogeneity of variance and normality of residuals (Kozak and Piepho, 2018).

## Results

### Seasonal periods

During the study period, black globe temperature at the Kaa gate reached a maximum of 51°C in December 2013 and dropped to as low as −9°C in July 2014. Rainfall totalled 301 mm during this 1-year study period, within the annual rainfall range of 250–350 mm (Fynn and Bonyongo, 2011). The hot seasons (hot-wet and hot-dry) were similarly hot (Table 1). Most rain occurred during the hot-wet season, but some rain fell during March (transition-dry season) and during November (hot-dry season; Table 1). The vegetation of the study area was greenest during the hot-wet and transition-dry seasons and least green during the hot-dry season (Table 1).

**Table 1:** Environmental conditions (mean ± SD) prevailing across four seasons during which gemsbok and wildebeest were free-living within the Bakgalagadi Schwelle, Botswana

	Hot-wet (Dec–Feb)	Transition-dry (Mar–May)	Cold-dry (Jun–Aug)	Hot-dry (Sep–Nov)
Black globe temperature (°C)				
Mean 24-h	27.8 ± 1.7	20.7 ± 4.0	14.1 ± 2.1	25.1 ± 2.5
Maximum 24-h	42.7 ± 2.7	36.2 ± 3.4	31.6 ± 1.6	42.1 ± 2.8
Minimum 24-h	16.9 ± 0.6	9.4 ± 4.5	0.8 ± 1.4	9.8 ± 1.9
Total rainfall (mm)^a^	189	77	0	35
Number of rainy days	25	8	0	9
Vegetation greenness index (NDVI)^b^	0.241 ± 0.08	0.301 ± 0.06	0.190 ± 0.01	0.174 ± 0.00

Note: ^a^Rainfall data were obtained from Climate Hazards Group InfraRed Precipitation with Station data (CHIRPS) for the same location as the weather station (S 24.3451°, E 20.6049°). ^b^Vegetation greenness data were obtained from the MODIS Terra Daily NDVI dataset for the entire study area.

### Activity

**Figure 1:**
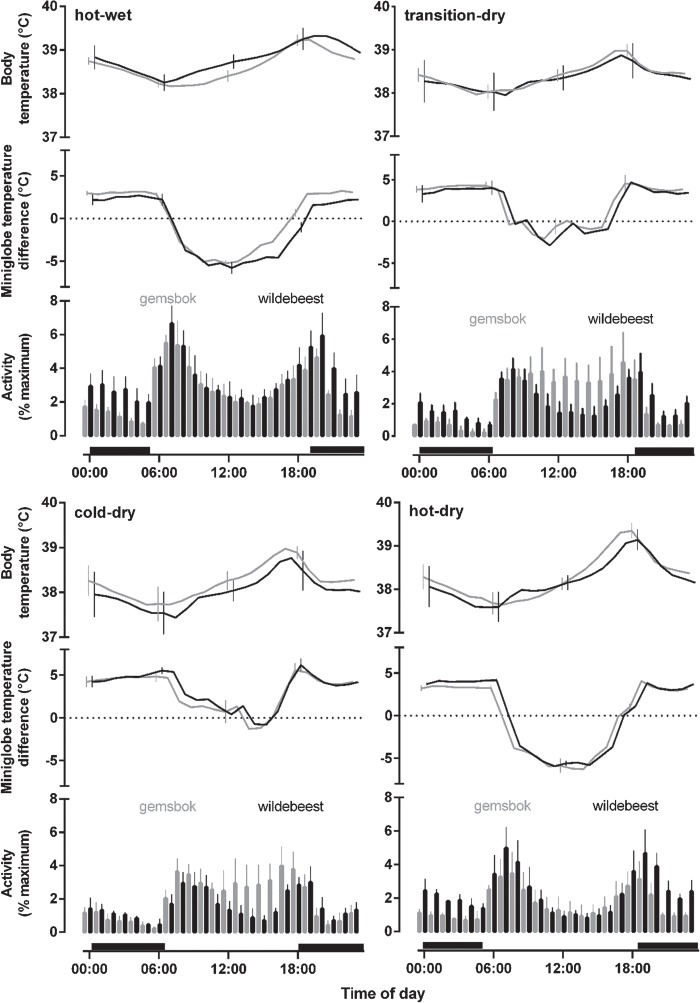
Diel rhythm (mean ± SD) of body temperature for gemsbok (*n* = 5, grey line) and wildebeest (*n* = 6, black line); microclimate selection for gemsbok (*n* = 5, grey line) and wildebeest (*n* = 2, black line); activity for gemsbok (*n* = 3, grey bars) and wildebeest (*n* = 3, black bars) comparing the four seasons, namely hot-wet, transition-dry, cold-dry and hot-dry. Microclimate selection is expressed as the difference between miniglobe temperature on the collar, i.e. at the site chosen by each individual and the temperature of an identical miniglobe exposed to the sun at a nearby reference miniglobe. Activity counts were expressed as a percentage of maximum counts for each logger. Black horizontal bars represent night.

Both species showed peaks of activity after dawn and around dusk and lowered activity during the heat of the day and at night. In gemsbok, these crepuscular peaks in activity were less pronounced in the transition-dry and cold-dry seasons compared to the other seasons because of an increase in activity during the middle of the day (Fig. 1).

**Figure 2:**
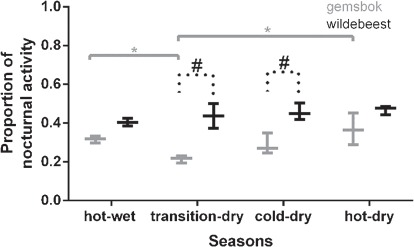
A boxplot showing the proportion of 24-h activity that occurred during the night (sunset to sunrise) for gemsbok (*n* = 3, grey bar) and wildebeest (*n* = 3, black bar) over the four seasons. The hashtags represent the difference between species, and significance bars represent the difference between seasons. ^*^P < 0.05.

The proportion of total 24-h activity taking place at night differed significantly between species (*F*_1,4_ = 29, *P* < 0.003; Fig. 2) and across seasons (*F*_3,12_ = 6.5, *P* = 0.006), with the two species responding differently to seasonal variation (*F*_3,12_ = 4.7, *P* = 0.02). Wildebeest were consistently more active nocturnally than gemsbok and significantly so during the transition-dry (*P* < 0.0001) and cold-dry (*P* = 0.01; Fig. 2) seasons. Gemsbok were more active nocturnally during the hot-wet and hot-dry seasons compared to the transition-dry season, whereas wildebeest showed no difference in their proportion of nocturnal activity across the seasons (Fig. 2).

### Microclimate selection

The intensity of shade selected by gemsbok and wildebeest did not differ between species (*F*_1,5_ = 0.41, *P* = 0.55; Fig. 1), but there were seasonal differences (*F*_3,15_ = 403, *P* < 0.0001), shown similarly by both species (*F*_3,15_ = 3.1, *P* = 0.06). The intensity of the shade selected was greater during the hot seasons than in the cooler transitional- and cool-dry seasons. During the hot seasons, both species selected microclimates that were ~10°C cooler than those recorded in the sun during the heat of the day on occasions. This difference was less during the cooler seasons, and both species consistently selected microclimates warmer than the reference globe during the night (Fig. 1). Both species spent 50–60% of the daylight hours in microclimates that were cooler than those experienced in the sun during both hot seasons, whereas during the cooler seasons they were seldom (transitional-dry: 23–30%; cold-dry: 17–18%) observed in cooler microclimates.

**Figure 3:**
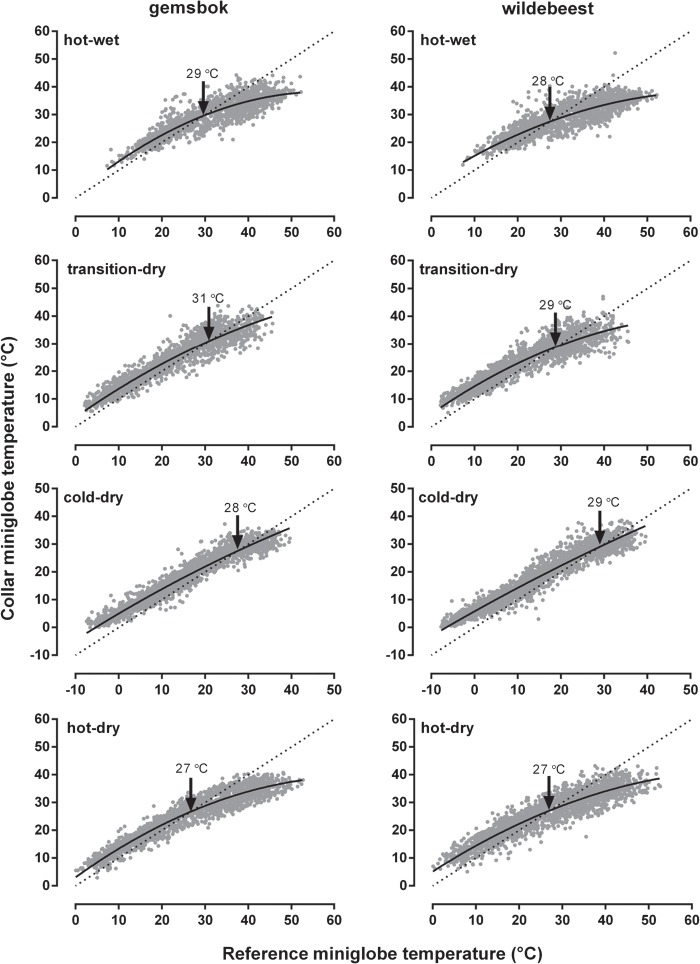
Relationship between miniglobe temperatures experienced by a representative female gemsbok (left-hand panels) and a representative female wildebeest (right-hand panels) and the reference miniglobe temperatures recorded across the four seasons. The dashed line is the line of identity (*y* = *x*), the solid black line is the non-linear polynomial regression. The arrow indicates the threshold at which the regression line crossed the line of identity.

There was no difference in the threshold temperature at which shade was selected between wildebeest and gemsbok (*F*_1,5_ = 0.6, *P* = 0.48), but there were seasonal differences (*F*_3,15_ = 5.3, *P* = 0.01). The threshold for seeking shade was lower during the hot-dry season (~27°C) compared to the other seasons (hot-wet: 28–29°C; transitional-dry: 29–31°C; cold-dry: 28–29°C; Fig. 3). There was no interaction between species and seasons (*F*_3,15_ = 2.3, *P* = 0.12).

### Distance to water

**Figure 4:**
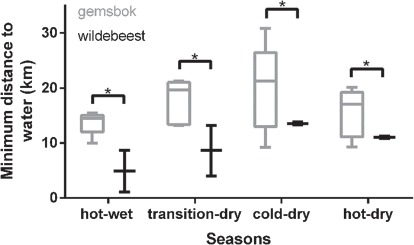
A boxplot showing the minimum 24-h distance to potential surface water for gemsbok (*n* = 5, grey bar) and wildebeest (*n* = 2, black bar) within the Bakgalagadi Schwelle, Botswana, over four seasons. The horizontal bars represent significant differences between species. ^*^P < 0.05.

The minimum distance to potential surface water over 24-h differed between species (*F*_1,5_ = 7.4, *P* = 0.04; Fig. 4). Wildebeest were situated closer to water than gemsbok on average across all seasons. However, there were only weak differences across seasons (*F*_3,15_ = 2.9, *P* = 0.07) and no interaction between species and season (*F*_3,15_ = 0.36, *P* = 0.78; Fig. 4).

**Figure 5:**
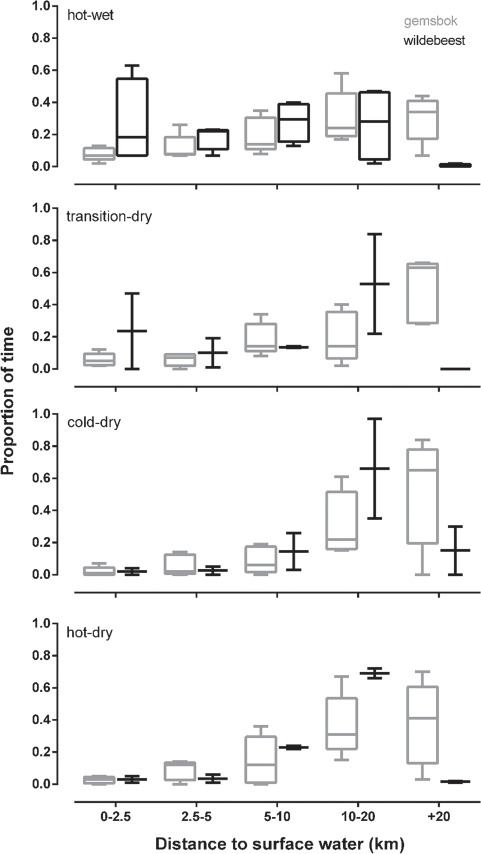
A boxplot showing the proportion of days per season in which gemsbok (*n* = 5, grey bar) and wildebeest (*n* = 2, black bar) were within five distance to water categories, 0–2.5, 2.5–5, 5–10, 10–20 and more than 20 km.

Wildebeest were located further than 10 km of a potential water source on almost 30% of the days during the hot-wet season, increasing to almost 70% by the hot-dry season (Fig. 5), but were seldom further than 20 km from water. In contrast, gemsbok were found >20 km away from the nearest water source on 40–60% of days across all seasons (Fig. 5).

### Body temperature

Throughout the year, body temperature generally varied daily between 37.5 and 39°C for both species. For gemsbok, an absolute minimum of 36.4 ± 0.7°C was recorded during the cold-dry season and a maximum of 40.7 ± 0.3°C during the hot-dry season. For wildebeest, an absolute minimum of 36.1 ± 0.5°C was recorded during the hot-dry season and a maximum of 41.2 ± 0.2°C during the hot-wet season (Fig. 1). Overall, mean (*F*_1,9_ = 0.17, *P* = 0.69), maximum (*F*_1,9_ = 0.00, *P* = 0.99), minimum (*F*_1,9_ = 0.07, *P* = 0.80) and amplitude (*F*_1,9_ = 0.11, *P* = 0.75) of 24-h body temperature rhythm did not differ between the two species across the four seasons (Fig. 1).

**Figure 6:**
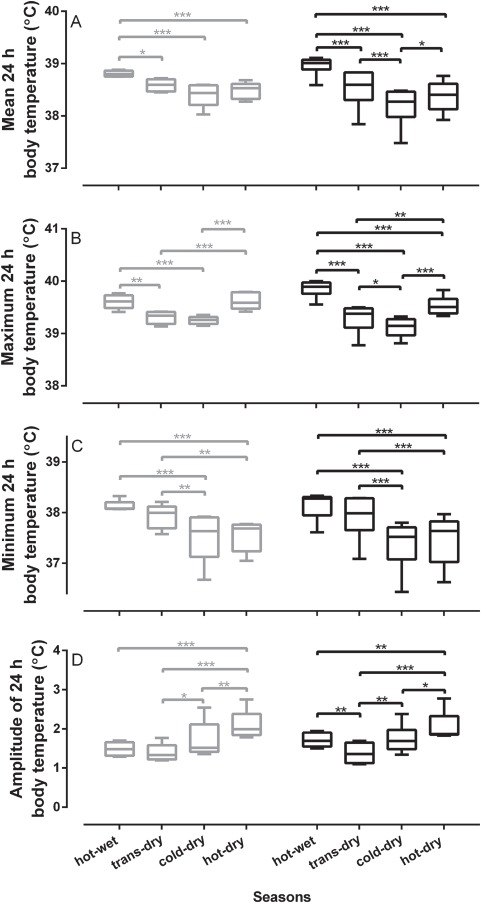
A boxplot showing the comparison of the profile of the 24-h body temperature rhythm between two sympatric species. (**A**) mean, (**B**) maximum, (**C**) minimum and (**D**) amplitude of the 24-h body temperature rhythm of gemsbok (*n* = 5, grey bar) and wildebeest (*n* = 6, black bar) over the four seasons. The horizontal bars represent significant differences between seasons. ^*^P < 0.05, ^**^P < 0.01, ^***^P < 0.001.

Mean (*F*_3,27_ = 52, *P* < 0.0001), maximum (*F*_3,27_ = 63, *P* < 0.0001), minimum (*F*_3,27_ = 39, *P* < 0.0001) and amplitude (*F*_3,27_ = 33, *P* < 0.0001) of 24-h body temperature rhythm did, however, differ seasonally (Fig. 6). Both species had higher mean 24-h body temperatures during the hot-wet season compared to the other seasons. The interaction between species and season (*F*_3,27_ = 4.9, *P* = 0.01) revealed that gemsbok mean 24-h body temperature remained fairly constant throughout the dry seasons, whereas wildebeest mean 24-h body temperature was ~0.3°C lower during the cold-dry season compared to the hot- and transitional-dry seasons (Fig. 6A). Maximum 24-h body temperatures of both species also responded differently across the seasons (*F*_3,27_ = 7.2, *P* = 0.001). Gemsbok maximum 24-h body temperatures were higher during both hot seasons compared to the cooler seasons, whereas wildebeest had the highest maximum 24-h body temperatures (39.9 ± 0.2°C) during the hot-wet season and the lowest maximum 24-h body temperatures (39.1 ± 0.2°C) in the cold-dry season compared to all the other seasons (Fig. 6B). Both species displayed lower minimum 24-h body temperatures during the cold-dry and hot-dry seasons compared to the hot-wet and transition-dry seasons, and there was no interaction between species and seasons (*F*_3,27_ = 0.57, *P* = 0.64; Fig. 6C). The low minimum 24-h body temperatures and high maximum 24-h body temperatures experienced during the hot-dry season resulted in both species displaying the largest amplitude of 24-h body temperature rhythm (gemsbok: 2.09 ± 0.4°C; wildebeest: 2.06 ± 0.4°C) during the hot-dry season compared to the other seasons (Fig. 6D). The 24-h amplitude of body temperature rhythm was lower during the transition-dry season compared to the cold-dry season for both species and compared to the hot-wet season for wildebeest, but there was no interaction between species and seasons (*F*_3,27_ = 1.4, *P* = 0.28; Fig. 6D).

## Discussion

This study documents the longest record of activity, microclimate selection and body temperature of two free-ranging sympatric species with differing water dependencies. Within typical rainfall conditions for the Kalahari, wildebeest and gemsbok showed fairly similar responses to seasonal variation in temperature and aridity. During hot times of the year, both species displayed crepuscular peaks in their activity rhythm and a substantial amount of nocturnal activity. They both maintained a fairly narrow rhythm of body temperature variation (homeothermy) through most of the year. Both species displayed slightly higher maximum 24-h body temperatures when conditions were hot and slightly lower minimum 24-h body temperatures when conditions were dry, resulting in the largest amplitude of 24-h body temperature rhythm during the hot-dry season.

During the hot seasons, gemsbok increased their proportion of nocturnal activity compared to the cooler seasons (Fig. 2), potentially compensating for activity lost while seeking shade to reduce high heat loads during the day. Similarly, sand gazelle (*Gazella subgutturosa marica*) and Arabian oryx (*Oryx leucoryx*) increased the proportion of nocturnal activity under hot and dry conditions within an Arabian Desert (Hetem *et al.*, 2012a), conditions even more extreme than those experienced within the Kalahari but in an area without predators. Wildebeest, on the other hand, consistently retained a fairly high level of nocturnal activity amounting to ~40% of their time. Similarly, wildebeest in the Central Kalahari Game Reserve did not increase nocturnal activity in the hot season (Selebatso *et al.*, 2017) and wildebeest in Etosha National Park spent 32% of their time foraging at night regardless of season (Berry, 1980).

During the hot seasons, both wildebeest and gemsbok selected microclimates (presumably shaded) that were at times 13°C cooler than the temperature of an identical miniglobe in the sun. Contrary to our expectations, water-dependent wildebeest did not seek shade at lower ambient temperatures or more frequently than the water-independent gemsbok, even during the hot-dry season. During the hot-dry season, both species sought cool microclimates when environmental heat loads exceeded 27°C. Other antelopes, including kudu (*Tragelaphus strepsiceros*; Hetem *et al.*, 2008), eland (Shrestha, 2012), Arabian oryx and sand gazelle (Hetem *et al.*, 2012b), similarly, sought shade between 28 and 30°C.

Within the Kalahari ecosystem, some pans retain water throughout the dry season, but animals may have to traverse large distances to reach them. Gemsbok were often more than 20 km away from potential water sources, a distance too far to travel to water and back during the course of a day. Wildebeest were generally closer to water than gemsbok, but nevertheless were frequently further than 10 km from surface water during the hot-dry season. In a similar environment in northern Botswana, wildebeest walked 20–40 km every 2–3 days to access water within the Boteti River during the dry season, a feat they achieved through highly efficient muscles that produce less heat per unit of energy consumed (Curtin *et al.*, 2018). Despite this, maximum 24-h body temperatures of wildebeest in our study area were highest during hot periods, potentially highlighting the trade-off of having to traverse large distances to access limited water sources.

During the hot-dry season when surface water in the Kalahari is most limited, both species showed high maximum 24-h body temperatures and low minimum 24-h body temperatures, resulting in the largest amplitude of 24-h body temperature rhythm (Fig. 6). The high maximum 24-h body temperatures could result from a combination of dehydration and exposure to high air temperatures, because ungulates appear to prioritize body water conservation over thermoregulation when they are water stressed (Fuller *et al.*, 2016; Hetem *et al.*, 2016; Hetem *et al.*, 2010). The low minimum 24-h body temperatures during the dry season may result from nutritional stress (Hetem *et al.*, 2016).

In general, both species responded remarkably similarly to seasonal variation in water and food resources. Only two other studies have compared body temperatures of sympatric ungulates inhabiting the same environment at the same time (Hetem *et al.*, 2012b; Shrestha *et al.*, 2012). Both studies found body temperature patterns to be remarkably similar between the species despite differences in body size (wildebeest, eland and impala (*Aepyceros melampus*; Shrestha *et al*., 2012); Arabian oryx and sand gazelle (Hetem *et al.*, 2012b)).

Both gemsbok and wildebeest showed little physiological stress under the prevailing environmental conditions for our study, as indexed by narrow body temperature rhythms (homeothermy). Water-dependent wildebeest may be able to maintain homeothermy despite high ambient temperatures because they were able to access water periodically to replenish water lost through evaporative cooling.

Our study was conducted in a region where there were no nearby fences restricting ungulate movements. In the Central Kalahari Game Reserve, to the east of our study area, wildebeest became prevented from moving westwards and northwards by a veterinary cordon fence. When artificially supplied water points were not maintained in the game reserve, some wildebeest travelled over 200 km to reach the nearest perennial water source to the south, while other wildebeest herds disappeared, never to return (Selebatso *et al.*, 2018). The wildebeest population in Botswana’s Kalahari region collapsed from well over 100 000 to a few thousands during severe drought conditions in 1983 when they moved eastwards seeking water and were trapped by the veterinary fence (Spinage, 1992).

Our study animals, both wildebeest and gemsbok, coped well with the near-average conditions prevailing during our study year despite being far from surface water for much of the time. Presumably the study region allowed them to obtain sufficient moisture from surface water and forage consumed, even during the hot-dry season. The situation may change when severe drought conditions ensue. During drought conditions, substantial mortality losses among wildebeest and eland (but not gemsbok) were recorded in the South African section of the Kalahari Transfrontier Park, to the south-west of our study area, when animals concentrated around the permanent waterholes (Knight, 1995), with adult males and calves being most susceptible. Thus, populations of these species would have retained the demographic resilience needed to promote recovery.

Thus, although artificial water points may need to be provided in some circumstances, they should be situated judiciously and only where essential (Owen-Smith, 1996). Excessive provision of water points led to crashes of numerous ungulate populations in private nature reserves bordering the Kruger National Park during the 1983 drought, due to widespread food depletion (Walker *et al.*, 1987). Water provision tends to favour the more common water-dependent grazers at the expenses of rarer antelope species (Harrington *et al.*, 1999). Our study has demonstrated some of the physiological mechanisms that enable gemsbok to thrive in the arid Kalahari despite restricted surface water availability and wildebeest to cope with conditions that are not too extreme.
